# Capsulectomy Can Successfully Treat Chronic Encapsulated Breast Seroma: A Case Report

**DOI:** 10.7759/cureus.21677

**Published:** 2022-01-27

**Authors:** Kjersti Fosheim, Sophie Bojesen, Hannah Troestrup, Anne-Vibeke Laenkholm

**Affiliations:** 1 Department of Surgical Pathology, Zealand University Hospital, Roskilde, DNK; 2 Department of Plastic Surgery, Zealand University Hospital, Roskilde, DNK

**Keywords:** case report, capsulectomy, refractory, seroma, breast

## Abstract

Chronic encapsulated seroma following breast cancer surgery is a rare entity, and management is challenging. We present clinical and pathologic findings in a patient with previous node-negative breast cancer and an extensive history of chronic bilateral seromas, successfully treated with capsulectomy. This is the first report of fibrous encapsulated breast seroma with bilateral presentation and late onset, following mastectomy with no prior axillary dissection. When managing breast seroma refractory to conventional treatment, the diagnosis of encapsulated seroma should be considered, followed by prompt capsulectomy.

## Introduction

Postoperative seroma is the generic term in common use for any abnormal subcutaneous accumulation of tissue fluid that may develop in preformed cavities, in response to surgical trauma [1,2]. It has been hypothesized that seromas are formed by fluid accumulation from disrupted blood and lymphatic vessels and/or acute inflammatory exudation [3-5]. The precise pathophysiological mechanisms underlying the development of seromas are only partly understood [3,4], making prevention and management difficult.

Seroma is not uncommon after breast cancer surgery, with a reported incidence of 3% to 90%, depending on diagnostic criteria and detection methods used [6]. Although often considered a minor postoperative sequela [7], seroma may delay recovery by causing skin necrosis, wound infection, and postponed adjuvant therapy [8], with potential negative impact on outcome and quality of life.

If allowed to persist, an encapsulated seroma may develop [9]. Few data exist as to the incidence, cause, and best ways to combat this rare phenomenon. While most postoperative breast seromas resolve spontaneously [5,10], or respond readily to conventional therapy (compression, aspiration, drainage) [7], treatment of encapsulated seroma is more complex (drain replacement, sclerotherapy, surgical intervention) [5,9,11-13]. Surgical capsulectomy may be required, as described in this case report.

## Case presentation

**Figure 1:**
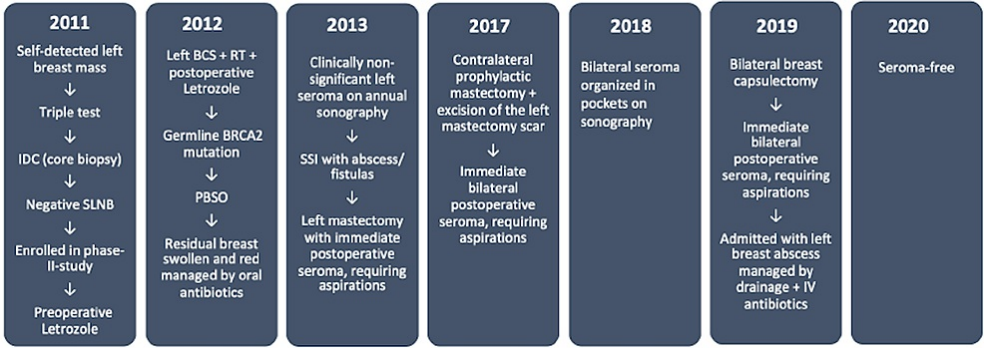
Timeline

A 71-year-old woman with previous breast cancer presented with a six-year history of refractory bilateral breast seromas, following sentinel lymph node biopsy (SLNB), endocrine therapy (ET), breast-conserving surgery (BCS), adjuvant radiotherapy (RT), and non-simultaneous double mastectomy (Figure 1).

The patient was diagnosed with early invasive cancer in the left breast in 2011. As part of a phase II study on preoperative neoadjuvant ET in postmenopausal women [14], SLNB was conducted within five months of primary surgery with excision of two negative sentinel nodes. According to guidelines, axillary dissection was not performed. The patient was assigned to receive initial ET for four months, followed by BCS, RT, and postoperative adjuvant letrozole for a total of five years. Pathologic evaluation of the lumpectomy specimen revealed a 20 mm, malignancy grade II invasive ductal carcinoma with clear margins and moderate residual disease, measured by the modified Miller-Payne grading system. The tumor was 100% ER positive with negative HER2 expression (score 1+) and a Ki-67 index of 1%. Postoperative recovery was without complications. 

The patient had a family history of breast and gynecological cancers, and in 2012 genetic testing identified a germline BRCA2 mutation. The same year she underwent risk-reducing bilateral salpingo-oophorectomy (PBSO), and in 2017 a risk-reducing contralateral mastectomy was performed. The patient had no prior history of breast augmentation.

Past medical history included hypertension, poorly controlled insulin-dependent type 2 diabetes mellitus, stage 3 chronic kidney disease, asthma, and previous tobacco use of 46 pack years. Body mass index (BMI) at the time of BCS was 31.

Clinical findings and diagnostic assessment

Five months after BCS, the patient presented with a sudden onset of swelling and redness of the skin of the residual breast with no history of trauma. Physical examination revealed diffuse erythema, warmth, edema with a “peau d’orange” appearance of the skin. No fluid collection was apparent. Body temperature and laboratory findings were normal. Seroma formation, infection, late post-surgical-/-radiation complications, as well as local cancer recurrence were considered. The condition improved with oral antibiotics, and no significant symptoms were apparent over the ensuing nine months.

**Figure 2:**
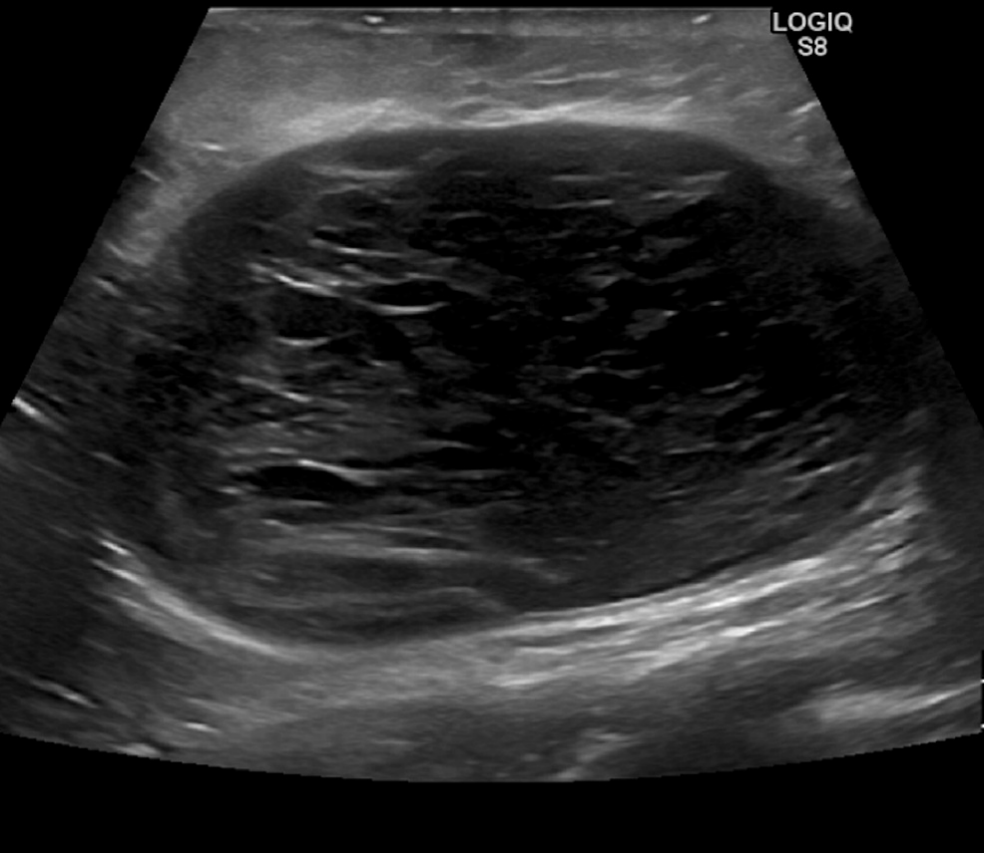
Breast Ultrasonography Image of a 15 x 10 x 5 cm breast seroma organized in pockets with hyperechoic capsule formation.

Thirteen months after primary surgery, the annual breast MRI with mammography revealed a small asymptomatic seroma and/or hematoma, but no local cancer recurrence. However, two months later the patient developed low-grade fever, malaise, fatigue, spontaneously draining breast abscess, and periareolar fistulas. Culture for bacterias showed minimal growth of normal skin flora. Ultrasonography revealed pockets of inflamed seroma with formation of a hyperechoic capsule (Figure 2). Oral antibiotics and compression bandaging proved ineffective.

Therapeutic interventions

In 2013, a simple left mastectomy was conducted to treat persistent breast infection, and in 2017, a contralateral prophylactic mastectomy with simultaneous asymmetric excision of the left mastectomy scar was performed. Each surgical procedure was followed by escalation of bilateral seroma formation. Seroma aspiration was initiated in 2013 and continued with varying frequency over a period of six years. Forty-three aspirations were required with a mean aspirated volume of 236 mL (range, 7-1250 mL). Cytologic analysis of the aspirated fluid composition was not performed.

**Figure 3:**
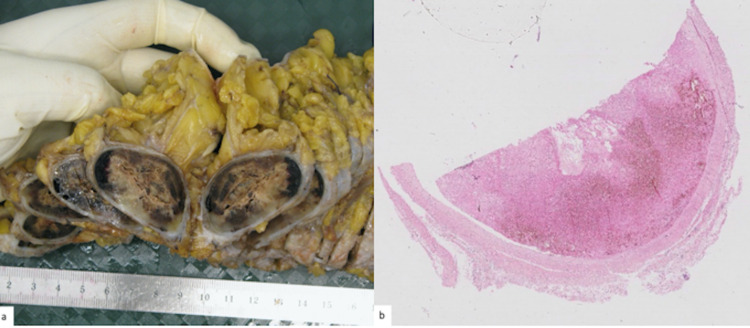
Fibrous Encapsulated Breast Seroma a. Gross appearance of the capsulectomy specimen demonstrates a distinct thick fibrous capsule surrounding seroma with hemorrhage. b. H&E staining demonstrates the thickened capsule and hematoma sequelae.

In 2019, a bilateral breast capsulectomy was performed. Fibrous capsules were identified and excised, and excess skin with overlying scarring was removed. Gross examination of both capsulectomy specimens revealed similar-looking bilateral changes with multiple communicating subcutaneous pockets surrounded by a distinct dense fibrous capsule. The pockets measured 25.7 cm in diameter, with up to 1.2 cm thick surrounding capsules (Figure 3a). Microscopically, a cavity with fibrinous-thread-containing material was observed surrounded by a peripheral well-defined dense fibrous capsule. The findings were consistent with bilateral organized encapsulated breast seroma (Figure 3b).

Surgeries (BCS, non-simultaneous bilateral mastectomy, bilateral capsulectomy) were performed by a number of experienced breast surgeons on an outpatient or short-stay basis. With the exception of BCS, surgical drains were used and removed when drainage volume reached < 50-100 mL/24 h. Early arm movements were encouraged, and rehabilitation therapy was initiated on the first postoperative day. Breast reconstruction was discussed, but was not recommended, due to obesity and coexisting comorbidities. Alternative therapy i.e. sclerotherapy (e.g. talc, tetracycline antibiotics, ethanol, erythromycin, fibrin glue) was not considered.

Follow-up and outcome

Four months after capsulectomy the patient was admitted with leukocytosis, fever, and an abscess in the left breast. The condition was successfully managed by drainage, intravenous antibiotics, and compression. No clinically relevant seroma was detected at nine-months’ follow up from the time of capsulectomy.

## Discussion

This case is believed to be the first presenting a breast cancer patient with excessive encapsulated seromas involving both breasts, requiring bilateral capsulectomy with no prior history of axillary dissection. Many patients develop transient low-volume seroma after surgery, but only few develop persistent refractory seroma. In this case, onset of symptomatic seroma occurred fifteen months from the time of primary surgery. Bilateral onset was seen years later following additional procedures.

Many efforts have been made in identifying potential patient- and therapy-related risk factors for seroma formation in breast surgery, but findings diverge [15]. Only a few predictors i.e. high BMI, diabetes mellitus, and poor health (American Society of Anesthesiologists score ≥ 3), have been established as significant predictors in a recent RCT [2], contradicting type of surgery as the principal factor of seroma formation [7,16,17]. Most other hypothesized seroma causes, i.e. age [2,6], hypertension [2,18], and smoking [6,19], have not been demonstrated consistently in the literature. Interestingly, mutated BRCA2 was a feature in this case, but could also be an incidental finding. To our knowledge, the significance of BRCA mutation carrier status on seroma formation is unknown. Thus, it remains unclear if the patient described was genetically predisposed to postoperative seroma, or if her unusual seroma course could have been predicted by a complex medical history with obesity and poor health. 

The impact of one’s surgical skills and duration of surgery on seroma formation have been thoroughly investigated. Some studies suggest that a surgeon’s experience and qualifications are preventive factors for the development of postoperative seroma [16,20], while others have found no such association [8]. These findings imply that seroma may be unavoidable despite impeccable surgical techniques.

Seroma has been shown to be significantly associated with surgical site infection (SSI) [17]. In the present case, SSI was diagnosed eight months prior to seroma onset, with subsequent recurrent infections. A critical review of the patient’s medical record revealed a clinically undetected seroma noted at the first annual breast MRI. An earlier and more intensive follow-up with prompt aspiration might have reduced the long-term risk of SSI, seroma recurrence, and fibrous encapsulation. On the other hand, this would have increased the risk of iatrogenic infection.

Several other potential seroma causative and protective factors have been investigated, i.e. surgical technique (conventional wound closure, flap fixation, quilting), postoperative management (drainage, compression therapy, rehabilitation), and the use of chemical agents (sclerosants). However, results are conflicting due to significant heterogeneity among studies [6], and further elaboration on these matters is beyond the scope of this report.

A few previous cases have described the successful effect of capsulectomy on post-axillary dissection encapsulated seroma [5,9,11-13]. However, it can be difficult to attribute seromas in the lateral chest site to the axillary versus the breast surgical procedure, because of the combined surgical field [17]. Because seroma can occur late, an extended follow-up is needed to detect all seroma recurrences, and hereby establish the true incidence of this postoperative sequela. The lack of seroma recurrence in previous reports is noteworthy and thus supports our observation of capsulectomy as an effective strategy in managing seroma refractory to other measures.

## Conclusions

This report highlights the benefit of capsulectomy as a treatment of persistent breast seromas proven resistant to compression and aspiration. Further research is required to determine the predictors of complex, treatment-refractory breast seromas and to identify effective strategies of prevention and management.
